# History of safe exposure and bioinformatic assessment of phosphomannose-isomerase (PMI) for allergenic risk

**DOI:** 10.1007/s11248-021-00243-0

**Published:** 2021-03-24

**Authors:** Rod A. Herman, Zhenglin Hou, Henry Mirsky, Mark E. Nelson, Carey A. Mathesius, Jason M. Roper

**Affiliations:** 1 Corteva Agriscience, 9330 Zionsville Road, Indianapolis, IN 47968 USA; 2 Corteva Agriscience, 8325 NW 62nd Avenue, Johnston, IA 50131 USA; 3 Corteva Agriscience, P.O. Box 30, Newark, DE 19714 USA

**Keywords:** Phosphomannose-isomerase, PMI, Selectable marker, Bioinformatics, Allergy, Safety

## Abstract

Newly expressed proteins in genetically engineered crops are evaluated for potential cross reactivity to known allergens as part of their safety assessment. This assessment uses a weight-of-evidence approach. Two key components of this allergenicity assessment include any history of safe human exposure to the protein and/or the source organism from which it was originally derived, and bioinformatic analysis identifying amino acid sequence relatedness to known allergens. Phosphomannose-isomerase (PMI) has been expressed in commercialized genetically engineered (GE) crops as a selectable marker since 2010 with no known reports of allergy, which supports a history of safe exposure, and GE events expressing the PMI protein have been approved globally based on expert safety analysis. Bioinformatic analyses identified an eight-amino-acid contiguous match between PMI and a frog parvalbumin allergen (CAC83047.1). While short amino acid matches have been shown to be a poor predictor of allergen cross reactivity, most regulatory bodies require such matches be assessed in support of the allergenicity risk assessment. Here, this match is shown to be of negligible risk of conferring cross reactivity with known allergens.

## Introduction

Phosphomannose-isomerase (PMI) has been expressed in commercialized genetically engineered (GE) crops as a selectable marker since 2010 (https://www.nature.com/articles/nbt.1842.pdf?origin=ppub), and GE events expressing the PMI protein have been approved globally by regulatory authorities (https://www.isaaa.org/gmapprovaldatabase/gene/default.asp?GeneID=37&Gene=pmi). The selectivity of this marker is enabled through the ability to derive energy from mannose by plant cells when they are tissue cultured on media where mannose is the predominant energy source (Negrotto et al. 2000). Recently, PMI was used as a selectable marker in the creation of event DP-915635–4 maize which is currently under development. In addition to the PMI protein, event DP-915635–4 maize expresses the IPD079Ea protein for control of corn rootworm (*Diabrotica* spp*.*) (Allen et al. 2018) and expresses the phosphinothricin acetyltransferase (PAT) protein for tolerance to glufosinate herbicide (Hérouet et al. 2005).

Since allergy is typically exhibited to a small subset of proteins, part of the safety assessment of GE crops includes an allergenicity assessment of any newly expressed proteins. This assessment uses a weight-of-evidence approach. Two key components of this allergenicity assessment include any history of safe human exposure to the protein and/or the source organism from which it was originally derived, and bioinformatic analysis identifying amino acid sequence relatedness to known allergens (Herman and Ladics 2018). These highly conservative bioinformatic criteria/thresholds for assessing allergenic risk were developed almost 20 years ago and are incorporated into most government regulatory guidance (FAO/WHO 2001; Ladics et al. 2011). Here the relevance of an eight-amino-acid contiguous match between the PMI protein and a frog allergen is evaluated in relation to potential cross reactivity to this protein.

## History of safe exposure

Although less common in plants, PMI proteins are produced by many organisms including those found in the human gut microbiome, and the history of safe use of this protein has been reviewed previously (Delaney et al. 2008). The source organism for the PMI protein as expressed in GE crops is *Escherichia coli* for which no natively expressed allergens are known (https://comparedatabase.org/). PMI has also been incorporated into multiple commercialized GE maize events and breeding stacks (cultivated since 2010), and also into high vitamin-A rice (event IR-00GR2E-5; “Golden Rice”) (https://www.isaaa.org/gmapprovaldatabase/gene/default.asp?GeneID=37&Gene=pmi), the latter of which has thus far received regulatory food, feed, and/or cultivation approval in Australia, Canada, New Zealand, The Philippines, and The United States (https://www.isaaa.org/gmapprovaldatabase/event/default.asp?EventID=528&Event=GR2E).

Maize events expressing the PMI protein have been grown widely with no known reports of adverse health effects including allergic responses (Su et al. 2020). Exposure for a decade to a widely cultivated crop (maize) expressing PMI with no reports of allergy provides additional strong evidence for the safety of the PMI protein.

## Bioinformatic assessment

### Sliding-window search

A threshold of concern for assessing allergenic risk where > 35% identity exists over an alignment of ≥ 80 amino acids was established almost two decades ago (FAO/WHO 2001). It is noteworthy that, while this threshold is very sensitive to cross-reactive risk, it also produces a high rate of false detections (Herman and Song 2019, 2020). Using this approach to search alignments between the PMI amino acid sequence and the allergen sequences in the 2020 COMPARE allergen database (https://comparedatabase.org/) identified no above-threshold matches.

### Eight-amino-acid contiguous match

**Fig. 1:**
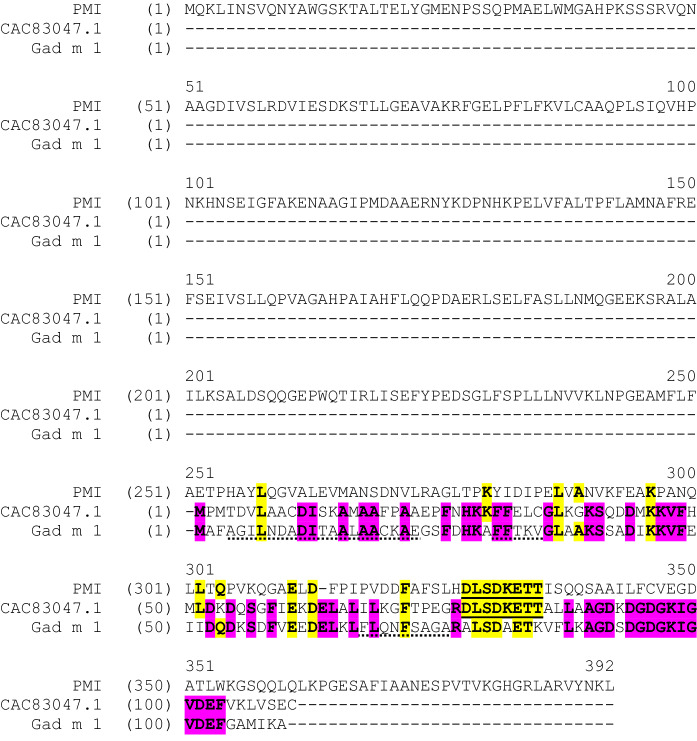
Amino acid alignment between PMI in DP-915635–4, CAC83047.1, and Gad m 1. Bolded amino acids are shared by two or more of the sequences. Yellow designates alignment between PMI and frog (CAC83047.1) and/or fish (Gad m 1) sequence. Purple designates alignment between frog and fish sequences but not PMI. Solid underline designates 8-mer alignment between PMI and frog sequence. Dotted underline designates IgE epitopes in fish sequence from Sánchez et al. (2016). (Color figure online)

An additional threshold of concern for assessing allergenic risk was established where a newly expressed protein in a GE crop shares an eight-amino-acid contiguous match with a known allergen based on the minimum size of an IgE epitope (Kleter and Peijnenburg 2004; Taylor 1997). There is a single eight-amino-acid contiguous match between the PMI selectable marker in event DP-915635–4 and the CAC83047.1 parvalbumin frog allergen (Fig. 1). As indicated earlier, PMI, as expressed in event DP-915635–4 maize, has a history of safe use in crops with no known adverse responses including allergy (Delaney et al. 2008). Frog allergy is rare and is a response to a parvalbumin that cross reacts with other parvalbumins found in fish that are the cause of more common fish allergies (due to shared cross-reactive IgE epitopes between the proteins) (Arif and Hasnain 2010; Hilger et al. 2004). All four known frog allergens (all parvalbumins) were first entered into the AllergenOnline database in 2007 (http://www.allergenonline.org/), the primary source used by technology developers for bioinformatic searches at that time, and were subsequently added to the COMPARE allergen database (https://comparedatabase.org/) upon its inception in 2017; therefore, these frog allergens would have been considered in the bioinformatic results supporting allergenicity assessments indicating negligible risk.

Although the IgE epitopes in frog parvalbumin have not been mapped, the eight contiguous amino acid match between PMI and the frog allergen CAC83047.1 is outside the epitopes identified in cross-reactive fish parvalbumins (Sánchez et al. 2016) (Fig. 1). The eight-amino-acid contiguous match between PMI and the CAC83047.1 frog parvalbumin has three mismatches with the homologous alignment in fish parvalbumin Gad m 1 (Sánchez et al. 2016). Further, these mismatches are dissimilar amino acid substitutions (A → D, A → K, and K → T) making this eight-amino-acid contiguous match highly unlikely to be a cross-reactive IgE epitope (Fig. 1). The IgE reactivity to fish parvalbumin is also largely governed by amyloid protein folds related to homology among these cross-reactive parvalbumins (Sánchez et al. 2016) that is not aligned with the overall PMI amino acid sequence.

In addition, there are no known cross-reactive allergens that share an eight-amino-acid contiguous match without also sharing significant homology over a much longer stretch of amino acids (e.g., > 35% identity over at least 80 amino acids) so such matches all appear to be false positives (Herman et al. 2009, 2021). The maximum identity over a sliding window of 80 or greater amino acids between PMI and the frog allergen (CAC83047.1) is 20% and such alignments do not include the eight-amino-acid contiguous match (alignments including the eight-amino-acid contiguous match have a maximum of 18.75% identity over 80 amino acids) which is substantially below the minimum identity observed for cross-reactive allergens (> 35%). In conclusion, the eight-amino-acid contiguous match between the PMI selectable marker in event DP-915635–4 maize and a frog allergen (CAC83047.1) is unlikely to represent a cross-reactive risk, which is supported by PMI’s history of safe use in crops.

### Detailed overall protein structures

**Fig. 2:**
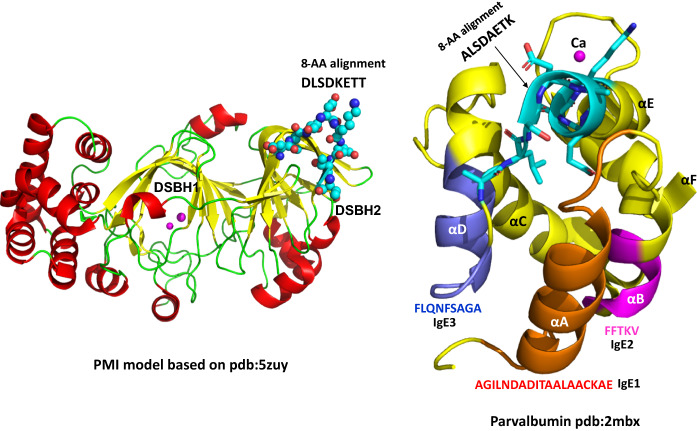
Three-dimensional structural comparison of PMI and fish parvalbumin. In the cartoon representation, cylindrical spiral ribbons stand for α-helices, arrows for β-strands, and linking ropes for loops. The left panel is a PMI model built with Swiss-Model (https://swissmodel.expasy.org/) based on PDB:5zuy. The model has good quality scores, GMQE:0.95 and QMEAN:0.53, due to its high sequence identity (86.2%) with the template. The two β-barrel domains are labeled as double-stranded β-helix (DSBH). The eight-amino-acid alignment is depicted as sticks-and-balls. The right panel shows a fish parvalbumin structure PDB:2mbx with three EF-hand or helix-loop-helix motifs, αA—αB, αC—αD, and αE—αF. The αC—αD and αE—αF structures containing bound Ca2+. The three IgE binding epitopes are highlighted in red on αA, magenta on αB, and blue on αD. The eight-amino-acid alignment is shown as sticks on αE. (Color figure online)

PMI and the frog parvalbumin have no meaningful homologous relationship either in sequence or at the three-dimensional structural level. These two proteins exhibit two distinct protein folding patterns (http://scop.mrc-lmb.cam.ac.uk/); PMI is a typical member of the cupin superfamily dominated by two neighboring β-barrels (Dunwell et al. 2001) while the frog parvalbumin and its close homolog, the fish allergen Gad m 1, consist of three tandem calcium-binding EF-hand motifs with a helix-loop-helix topology (Moraes et al. 2014) (Fig. 2). The matched eight contiguous amino acid peptides are located in different structural environments within their respective proteins and assume distinct conformations in the two proteins; In PMI, the sequence DLSDKETT adopts a loop and β-strand in the second double-stranded β-helix barrel while the matched frog peptide is part of the αE helix. The IgE epitopes of Gad m 1 have been identified with peptide scanning (Sánchez et al. 2016). Mapping of the epitopes onto the Gad m 1 structure shows no spatial overlap of the eight-amino-acid peptide match DLSDKETT on αE with the IgE epitopes AGILNDADITAALAACKAE on αA, FFTKV on αB, or FLQNFSAGA on αD (Fig. 2).

Previous studies have demonstrated that parvalbumin structural integrity is important for its allergenicity. In parvalbumin, a bound Ca2+, occupying the loop eye in helix-loop-helix motif, plays a central role in structural stability. First, Ca2+ depletion was shown to reduce IgE binding to parvalbumin (Bugajska-Schretter et al. 1998). In addition, a hypoallergenic recombinant parvalbumin produced as a subcutaneous immunotherapeutic, with a mutation abolishing its calcium binding capacity, exhibited an altered structural conformation and had a 95% reduced IgE reactivity (Swoboda et al. 2007; Zuidmeer-Jongejan et al. 2015). This clearly indicates that the structural conformation and geometrical relationship among identified fish allergen epitopes are crucial for their allergenicity, but a similar allergenic location near the matched eight-amino-acid peptide does not exist in PMI due to its distinct structure. Thus, the eight-amino-acid contiguous match does not suggest any similar allergenic activity for PMI.

## Conclusion

PMI has been expressed in commercialized GE crops since 2010, and the presence of an eight-amino-acid contiguous match between PMI and a frog allergen has been known for more than a decade and considered a negligible risk by developers and global regulatory experts (https://www.isaaa.org/gmapprovaldatabase/gene/default.asp?GeneID=37&Gene=pmi). As previously discussed, short amino acid matches have been shown to be a poor predictor of allergen cross reactivity (Herman et al. 2009, 2021). However, the bioinformatic rationale supporting this negligible risk for PMI has not been previously summarized in the peer-reviewed scientific literature. The lack of sufficient similarity between the PMI and the CAC83047.1 parvalbumin frog protein to enable IgE cross reactivity is consistent with the negligible risk of cross reactivity supported by the history of safe exposure to PMI in commercialized GE crops.
